# Truncating Homozygous Mutation of Carboxypeptidase E (*CPE*) in a Morbidly Obese Female with Type 2 Diabetes Mellitus, Intellectual Disability and Hypogonadotrophic Hypogonadism

**DOI:** 10.1371/journal.pone.0131417

**Published:** 2015-06-29

**Authors:** Suzanne I. M. Alsters, Anthony P. Goldstone, Jessica L. Buxton, Anna Zekavati, Alona Sosinsky, Andrianos M. Yiorkas, Susan Holder, Robert E. Klaber, Nicola Bridges, Mieke M. van Haelst, Carel W. le Roux, Andrew J. Walley, Robin G. Walters, Michael Mueller, Alexandra I. F. Blakemore

**Affiliations:** 1 Section of Investigative Medicine, Division of Diabetes, Endocrinology, and Metabolism, Department of Medicine, Imperial College London, London, United Kingdom; 2 Imperial Centre for Endocrinology, Imperial College Healthcare NHS Trust, Hammersmith Hospital, London, United Kingdom; 3 Centre for Neuropsychopharmacology and Computational, Cognitive and Clinical Neuroimaging Laboratory, Division of Brain Sciences, Department of Medicine, Imperial College London, Hammersmith Hospital, London, United Kingdom; 4 Metabolic and Molecular Imaging Group, MRC Clinical Sciences Centre, Imperial College London, Hammersmith Hospital, London, United Kingdom; 5 Centre for Cardiovascular Genetics, UCL Institute of Cardiovascular Science, London, United Kingdom; 6 NIHR Imperial BRC Genomics Facility, Faculty of Medicine, Imperial College London, London, United Kingdom; 7 NW Thames Regional Genetics Service, Kennedy Galton Centre, North West London Hospitals NHS Trust, Northwick Park Hospital, Harrow, United Kingdom; 8 Department of Paediatrics, Imperial College Healthcare NHS Trust, St Mary's Hospital, London, United Kingdom; 9 Department of Paediatric Endocrinology, Chelsea and Westminster Hospital, London, United Kingdom; 10 Department of Medical Genetics, Wilhelmina Children’s Hospital, University Medical Center Utrecht, Utrecht, The Netherlands; 11 Diabetes Complications Research Centre, Conway Institute, School of Medicine and Medical Science, University College Dublin, Dublin, Ireland; 12 Department of Epidemiology and Biostatistics, School of Public Health, Imperial College London, London, United Kingdom; 13 Clinical Trial Service Unit and Epidemiological Studies Unit (CTSU), Nuffield Department of Population Health, University of Oxford, Oxford, United Kingdom; INSERM, FRANCE

## Abstract

Carboxypeptidase E is a peptide processing enzyme, involved in cleaving numerous peptide precursors, including neuropeptides and hormones involved in appetite control and glucose metabolism. Exome sequencing of a morbidly obese female from a consanguineous family revealed homozygosity for a truncating mutation of the *CPE* gene (c.76_98del; p.E26RfsX68). Analysis detected no CPE expression in whole blood-derived RNA from the proband, consistent with nonsense-mediated decay. The morbid obesity, intellectual disability, abnormal glucose homeostasis and hypogonadotrophic hypogonadism seen in this individual recapitulates phenotypes in the previously described *fat/fat* and *Cpe* knockout mouse models, evidencing the importance of this peptide/hormone-processing enzyme in regulating body weight, metabolism, and brain and reproductive function in humans.

## Introduction

An unknown proportion of severe cases of obesity are caused by monogenic disease. Many of the known monogenic forms of obesity, affecting appetite regulation through hypothalamic pathways, including the leptin-melanocortin pathway, were first identified from murine models of obesity. It is now two decades since the discovery of defects in *Lep* and *Lepr* in the mouse models *ob/ob* and *db/db* respectively led to the discovery of the first monogenic obesity syndromes, leptin and leptin receptor deficiency, in humans [1, 2]. The more recently reported mutations in SH2B1 causing obesity and maladaptive behaviours also followed on from investigation of the severely obese *sh2b1*-null mice models [3]. Another spontaneously occurring mutation causing murine obesity is the *fat/fat* mouse. CPE was discovered in 1982, and mutations in the *Cpe* gene causing the *fat/fat* phenotype have been known since 1995 [4, 5]. However, no null mutations in *CPE* have been described in humans to date. Carboxypeptidase E (CPE) is involved in the processing of the majority of neuropeptides and peptide hormones, removing C-terminal basic residues following initial cleavage by an endopeptidase [6]. Absence of functional CPE in the *fat/fat* mouse and *Cpe* knockout mouse leads to abnormally low levels of a number of neuropeptides and peptide hormones resulting in a range of phenotypes, including late-onset obesity, hyperproinsulinaemia, infertility, anxiety and depression, hippocampal neuronal degeneration and memory deficits [4, 7–11].

Recently, exome sequencing of severely obese individuals has been instrumental in identifying several new forms of monogenic obesity [3, 12–15]. Here we present the results of whole exome sequencing of a consanguineous Sudanese family with a Mendelian pattern of a complex obesity syndrome, leading to the discovery of a new monogenic obesity syndrome, CPE deficiency, in a morbidly obese woman with intellectual disability, type 2 diabetes mellitus (T2DM) and hypogonadotrophic hypogonadism, recapitulating the phenotype of the *fat/fat* mouse.

## Methods

### Study participants

In this study we investigated a morbidly obese Sudanese female proband and her family, recruited from the adult genetic obesity clinic run by APG at Hammersmith Hospital, Imperial College Healthcare NHS Trust, London UK. Whole blood samples were taken from 6 members of the family for DNA extraction. All subjects gave written informed consent for participation in this study. The study was specifically approved by the National Research Ethics Service Committee London – West London (study number 12/LO/0396) and National Research Ethics Service Committee London—Fulham (study number 07/Q0411/19). The individuals described in this manuscript have given written informed consent (as outlined in the PLOS consent form) to publish these case details.

### Exome sequencing and variant calling

**Fig 1:**
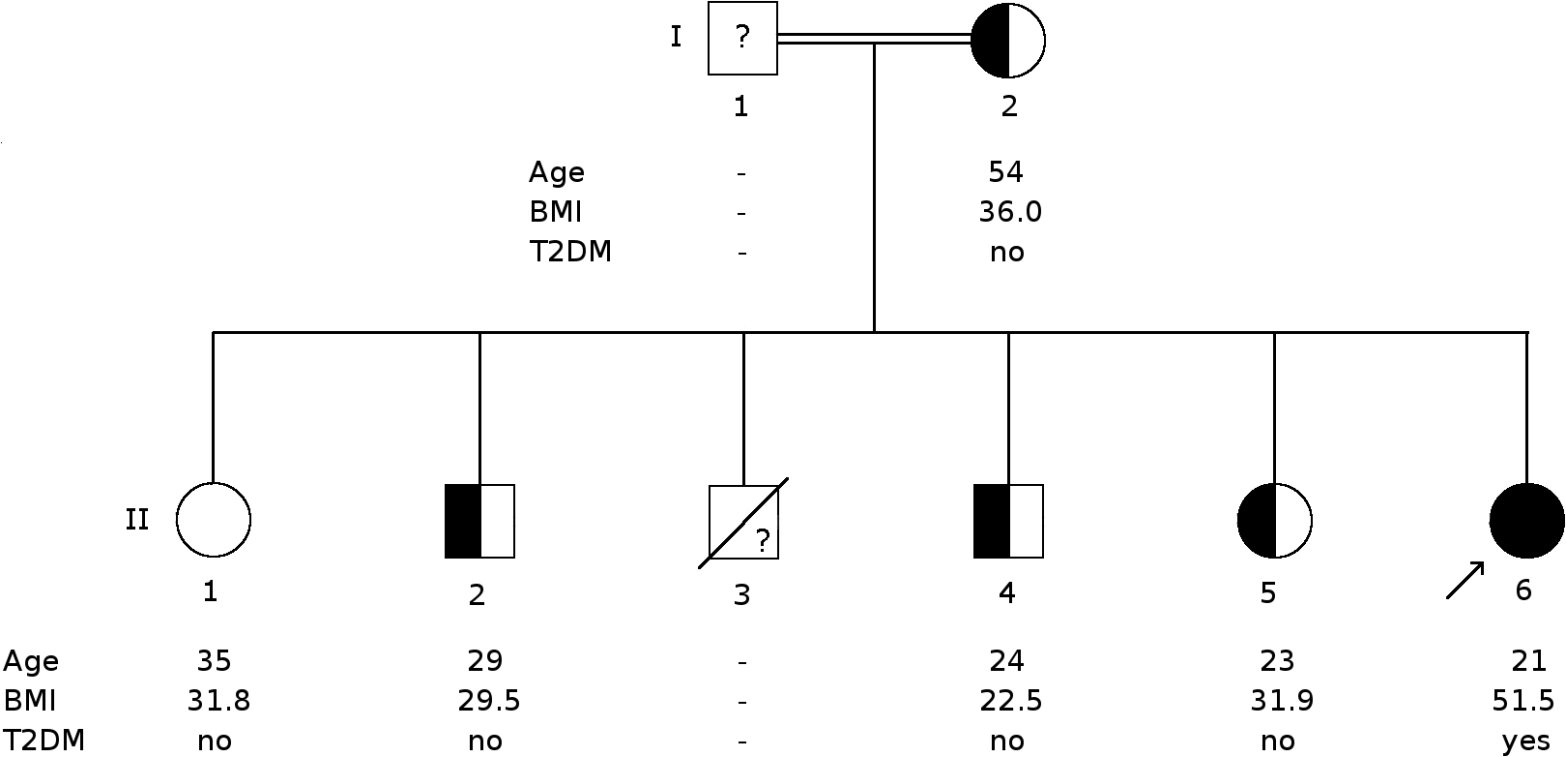
Pedigree of the affected family. Circles represent females and squares represent males. The proband is indicated by an arrow. Solid symbols indicate homozygosity for E26RfsX68, while half solid symbols indicate heterozygosity and open symbols non-carriers. The question mark in I.1 and II.3 indicate that genotype is not known. BMI, body mass index, in kg/m^2^, and age are as of day of examination. T2DM, Type 2 diabetes mellitus.

For the proband, mother and one sister (II.6, I.2 and II.5 respectively in Fig 1), whole-exome sequencing libraries were prepared using SureSelectXT Human All Exon V4+UTRs (71Mb) (Agilent Technologies, Santa Clara, CA) and sequenced on a HiSeq25000 platform generating 100bp paired end reads (performed by the Genomics Laboratory, MRC Clinical Sciences Centre, Imperial College London, UK). The quality of sequencing data was assessed with FastQC version 0.10.0. BWA mem version 0.7.2 was used to map sequencing reads to the GRCh37 (hg19) reference assembly of the human genome. To reduce false positive read mapping the hs37d5ss decoy sequences obtained from the 1000 genomes project FTP server were included as mapping targets. After reference mapping, duplicate reads were marked with Picard tools version 1.85. Processing of mapped reads and calling of single nucleotide variants and short insertions/deletions was carried out with the Genome Analysis Toolkit (GATK) version 2.6. Copy number variation (CNV) analysis was carried out in the proband, and her mother and sister (II.5), by read depth analysis of exome sequencing data [16].

### Variant prioritisation

Based on a family history of consanguinity (the proband’s parents are first cousins) and the extreme phenotype of the proband, an autosomal recessive mode of inheritance was hypothesised. Therefore all homozygous or compound heterozygous exonic variants, not present in a homozygous state in the unaffected mother and sister and at a read depth of at least 5 were considered. These variants were prioritised based on minor allele frequency of <1% in the 1000 Genomes project phase 1 release and the NHLBI Exome Sequencing Project (NHLBI Esp) [17, 18]. Variants that were synonymous or predicted to be benign by two out of three *in silico* prediction programs (SIFT, Polyphen2, PROVEAN1) were excluded [19, 20].

A list of genes known to cause obesity when disrupted and a similar list for intellectual disability were curated based on Online Mendelian Inheritance in Man (OMIM) and HGMD Pro database searches and literature (S1 and S2 Tables). All variants found by whole exome sequencing in the subjects (I.2, II.5 and II.6) were screened against the lists to exclude known genetic causes of obesity and intellectual disability.

### Variant validation and segregation analysis

The deletion found in CPE was validated through Sanger sequencing. Primers used to amplify the first exon of CPE were CPE_F1: GGAAGGTGAGGCGAGTAGAG and CPE_R1: CCCTTACCAGGCTCATGGAC. Because of the high GC-content of the region a denaturation temperature of 98°C was used. The same method was used to determine the segregation of the mutation among the family members.

### Expression analysis

Real time PCR analysis of *CPE* mRNA expression was performed in blood samples from the proband (II.6), heterozygous sister (II.5) and six control females matched for age, BMI and T2DM status. Total RNA was isolated from whole blood samples using the PAXgene blood RNA kit (Qiagen Ltd, Manchester, UK). Reverse transcription to obtain cDNA was carried out with 500ng total RNA using the RT^2^ Easy First Strand kit (Qiagen Ltd). Quantitative PCR was performed on each sample in triplicate, on a CFX384 real-time PCR detection system (Bio-Rad Laboratories, Hemel Hempstead, UK), using RT^2^ SYBR Green qPCR Mastermix, with primer assays for *CPE* (NM_001873, amplifies a 90bp product within exon 8 and 9) and the housekeeping gene *HPRT1* (NM_000194) (Qiagen Ltd). Relative expression levels for the proband, sibling and control samples were determined using the ΔΔCt method using a common reference sample, and are presented in as fold change in expression (2^(-ΔΔCt)^) [21].

## Results

### Clinical characterisation

**Fig 2:**
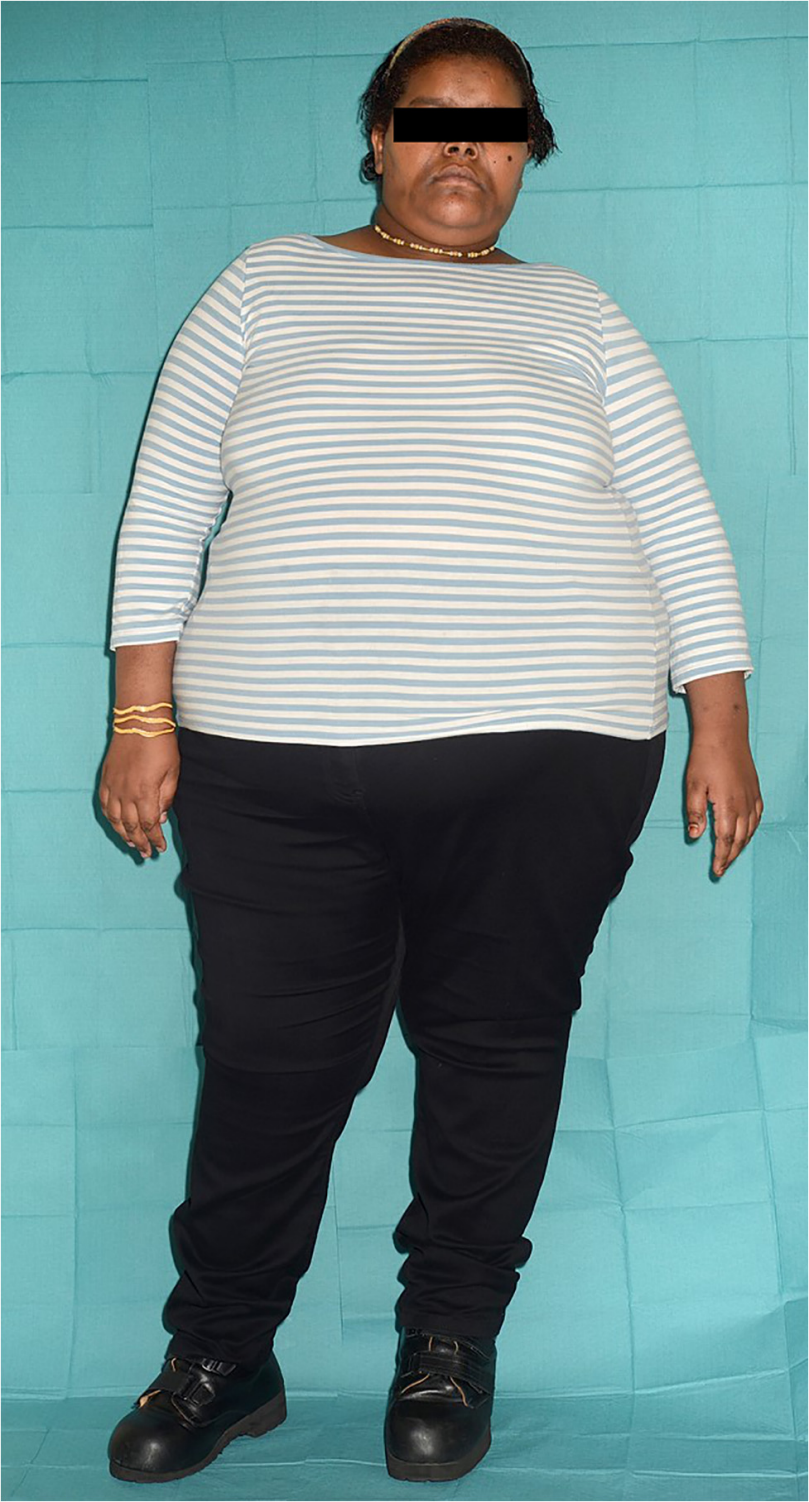
Clinical features of proband with homozygous truncating *CPE* mutation. Photograph of proband carrying a homozygous truncating deletion of *CPE*. Specific written consent for the photograph and case details was obtained from proband and mother. At the time of examination, the proband had a weight of 130.2 kg and height of 1.59 m with body mass index (BMI) 51.5 kg/m^2^. There was some intellectual disability, for example despite adequate schooling she was unable to read or write words. She had newly diagnosed type 2 diabetes mellitus with fasting glucose 383 mg/dL, 21.1 mmol/L; HbA1c 114 mmol/mol, 12.6%) and hypogonadotrophic hypogonadism with primary amenorrhea (serum oestradiol 78 pmol/L [post-menopausal range <100 pmol/L], 21.2 pg/mL; LH 2.7 IU/L, FSH 2.0 IU/L). Serum hormone analysis excluded other causes of amenorrhoea including polycystic ovary syndrome and hyperprolactinaemia (testosterone 1.2 nmol/L (normal <2.7), 0.35 ng/mL (normal <0.78); normal androstenedione, 17-hydroxyprogesterone, dehydroepiandrosterone sulphate (DHEAS), prolactin). There was no history of depression or anxiety.

The proband examined in this study was a 20 year old morbidly obese, Sudanese female, with childhood-onset obesity (current body mass index (BMI) 51.5 kg/m^2^), intellectual disability, newly diagnosed T2DM and hypogonadotrophic hypogonadism (Fig 2 and individual II.6 in Fig 1). An older brother (II.3), who died of unknown causes at the age of 21 years, also had childhood-onset severe obesity, intellectual disability and hypogenitalism, but no DNA was available from this individual. Other potential genetic causes of this phenotype, Prader-Willi syndrome and Fragile X syndrome, had previously been excluded in the proband by *SNRPN* DNA methylation analysis and demonstration of normal 5’-UTR CGG repeat number in the *FMR1* gene. No abnormality was detected by clinical array comparative genomic hybridisation (Agilent 8x60K 60mer oligo, ISCA design 024612). Other family members: the proband’ s mother; two sisters and two brothers (Fig 1), all had a history of mild obesity, with one brother (II.4) who achieved normal weight through lifestyle changes. There was no history of intellectual disability, amenorrhoea or T2DM in any of other family members. Although clinical data available from the father (I.1) revealed no obesity or any signs of intellectual disability, hypogonadism or T2DM, DNA was not available for further testing.

### Whole exome sequencing data analysis

**Fig 3:**
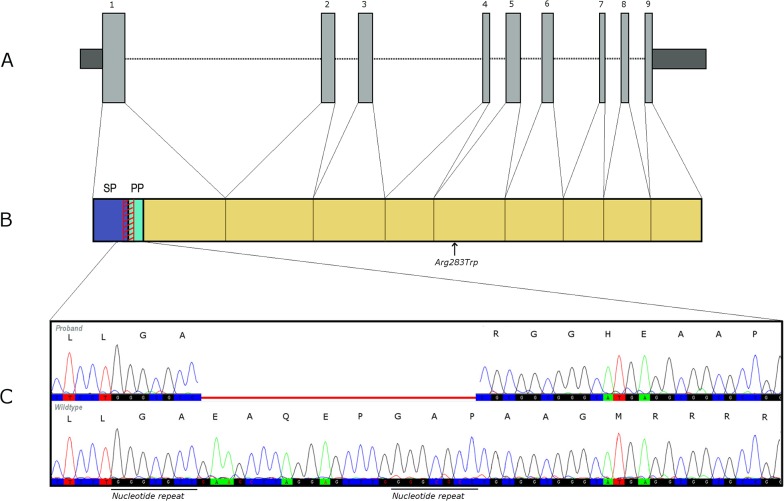
Location of c.76_98del; p. E26RfsX68 *CPE* mutation. A: Schematic overview of the exons of *CPE* (Refseq: NM_001873). Dark shaded areas are UTRs and light grey areas are coding regions. B: Human CPE protein (UniprotKB: P16870). Location of the E26RfsX68 mutation is shown by the red diagonally striped region. Arrow shows the location of Arg283Trp. SP, signalling peptide; PP, pro-peptide. C: Indicative chromatogram of the deletion in the proband and the normal wild-type sequence. The deletion is indicated in red. Amino acid changes caused by the frameshift are shown above the chromatogram.

After the application of the filtration strategy of the exome data, four homozygous, rare and predicted deleterious variants were found in the proband, and were in a heterozygous state or absent in the mother and heterozygous sister (Table 1). Only one of these was within a candidate gene for obesity: a frameshift deletion, c.76_98del, in exon 1 of the *CPE* gene, resulting in a p.E26RfsX68 truncation of the protein. An exact 7 nucleotide repeat (GGGCGCC) at the breakpoints, might indicate a microhomology-mediated deletion mechanism (Fig 3) [22].

**Table 1:** Homozygous mutations identified in proband.

Gene symbol	Variant	Exonic function	*in silico* prediction			1000 genome / ESP650	SNP 138	OMIM
			PolyPhen-2	SIFT	PROVEAN			
*CPE*	23_31del	Frameshift deletion	-	-	-	np	np	Less active protein leads to pre-disposition of early onset of T2DM
*MYL1*	M1fs	Frameshift insertion	-	-	-	np	np	-
*XDH*	L287V	Missense mutation	Damaging	Deleterious	Neutral	0.0002	rs138674014	Xanthinuria Type 1
*PABPC4L*	R263T	Missense mutation	-	-	-	np	np	-

Details of the homozygous, rare and predicted deleterious variants found in the proband, which were either absent or in heterozygous state in the mother and sister (II.5). np, not present in database; *CPE*, Carboxypeptidase E; *MYL1*, myosin, light chain 1; *XDH*, xanthine dehydrogenase; *PABPC4L*, poly(A) binding protein, cytoplasmic 4-like.

Sanger sequencing confirmed homozygosity for this mutation in the proband and heterozygosity in her mother, sister (II.5) and two unaffected brothers (II.2 and II.4). Another unaffected sister (II.1) did not carry the deletion (Fig 1). All other variants found in the proband, mother and sister (II.6, I.2 and II.5) by exome sequencing and the predicted CNVs were screened for known obesity and/or intellectual disability causing variants, but no variants were found that provided an explanation for the phenotypes.

As far as we are aware no *CPE* null mutations have been described in humans so far. The E26RfsX68 mutation has not been reported in publicly-available datasets from the 1000 Genomes project and the NHLBI Exome Sequencing Project. The deletion, however, is reported in two Caucasians in heterozygous state in the Exome Aggregation Consortium (ExAC) [23].

### CPE mRNA expression levels

Since the mutation found in *CPE* causes a frameshift and premature truncation of the protein, it is directly deleterious and likely to be silenced by nonsense-mediated decay. To confirm this, mRNA analysis was performed using real time PCR on the proband, a heterozygous sister (II.5) and 6 matched female controls (age range 32–59 years; BMI range 47.8–53.3 kg/m^2^; 3 with and 3 without T2DM).

**Fig 4:**
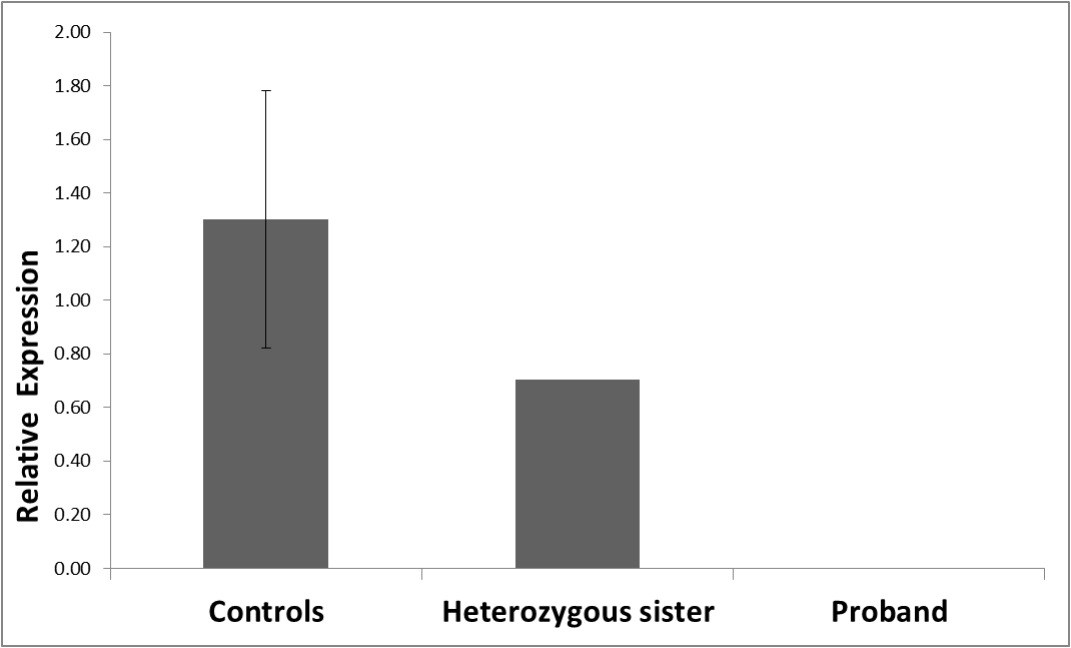
CPE mRNA expression levels. Real time PCR analysis of *CPE* mRNA expression in blood samples from the proband (II.6), heterozygous sibling (II.5) and six controls. For controls mean ± SEM (standard error of the mean) is depicted. All analyses were conducted in triplicate.

The overall mean coefficient of variation (CV) for Ct (threshold cycle) values of replicate samples (for which amplification products were obtained) was 2% for *CPE* and 1% for *HPRT1*, with a mean CT of 34.43 (SD = 1.21) for the *CPE* assay and a mean Ct of 29.15 (SD = 0.11) for the *HPRT1* assay. No *CPE* expression was detected in blood RNA from the proband after 40 cycles of amplification, while low but detectable levels were present in the six control samples and the reference sample. The value for normalised *CPE* expression in the heterozygous sibling was at the lower end of the range seen in the controls (Fig 4). Expression of the housekeeping gene *HPRT1* was detected in the proband, sister and all control samples, demonstrating that lack of detectable *CPE* expression in the proband was not due to insufficient or poor quality cDNA template. Ct values for the controls, patient, sibling and reference samples obtained for the *CPE* and *HPRT* assays and ΔΔCt values for all test samples are listed in S3 Table.

## Discussion

In this study we present a novel form of monogenic obesity in humans by identifying for the first time a homozygous deleterious mutation in *CPE*, leading to complete lack of its expression.

Although null mutations in *CPE* have never been reported in humans before, a heterozygous missense mutation (Arg283Trp) resulting in a less active enzyme was reported to affect age of onset of T2DM in specific Ashkenazi families, but unfortunately no details of BMI or obesity phenotypes were given [24]. In an earlier study, researchers screened for variants in *CPE* in a total of 269 Japanese subjects with non-insulin dependent T2DM, of whom 104 were also obese. However, no mutations affecting the coding region were found. [25] Common variants in *CPE* have not been associated with anthropometric or glycaemic traits in large-scale meta-analyses, but two intronic single nucleotide polymorphisms, rs1946816 and rs4481204, were recently reported to be associated with BMI in a European-American cohort [26]. The rare occurrence of heterozygous mutations and the absence of homozygous frameshift or stop-codon generating mutations in publicly available datasets (the 1000 Genomes project, the NHLBI Esp and ExAC) imply that disruption of *CPE* is uncommon. The recent finding of two Caucasians in the ExAC database heterozygous for exactly the same deletion as found in the Sudanese family described here, could point towards a hotspot for breakpoints leading to this mutation. The finding that in the family presented here, the deletion breakpoints are aligned with a nucleotide repeat (pointing towards a microhomology-mediated deletion mechanism, Fig 3) could indicate that this deletion may not be a unique occurrence. The high denaturation temperature that was needed to amplify the region for PCR, could explain why this mutation has not been seen before in large scale next generation sequencing cohorts, which is confirmed by a below average coverage of this region in the open databases available (data on sequencing quality and depth available from ExAC).

Beside the homozygous frameshift mutation in CPE, three other rare homozygous, predicted to be pathogenic mutations were found in the proband, but not or only in heterozygous state in the mother or sister (Table 1). However, all are less likely to contribute to the phenotype of obesity, T2DM, hypogonadotrophic hypogonadism or intellectual disability seen in the proband. *XDH*, encoding for Xanthine dehydrogenase, is associated with human disease according to OMIM (Online Mendelian Inheritance in Man) with deleterious mutations in *XDH* known to cause Xanthinuria type I. However, the proband had no history of kidney stones or renal failure and the mutation found (rs138674014) has so far not been linked to Xanthinuria type 1. Not much is known about the function of *PABPC4L*, besides its expression in the brain and multiple other tissues. A recent study on rare CNVs found an association, although not genome wide significant, between a deletion covering PABPC4L and treatment resistant depression. [27] The proband however does not have a history of depression. MYL1 encodes for a myosin alkali light chain active in embryonic, foetal and adult fast skeletal muscle [28]. The mutation found in the proband, a deletion of the first nucleotide of the coding region of MYL1, in first instance might appear to cause a frameshift starting from the first amino acid sequence, but the repeat of 10 similar nucleotides preceding the deletion in the non-coding region makes it less likely an actual frameshift will occur. Examination of this specific nucleotide repeat, preceding the coding region of MYL1, in the ExAC dataset, shows that variation in this region is not particularly rare (minor allele frequency up to 0.03041 across different populations).

*CPE* is a highly conserved gene, located on chromosome 4q32.3, and is widely expressed in human tissues, including neuropeptide-rich areas of the brain and endocrine tissues [29]. This is in line with the hormone/peptide-processing function of CPE in endocrine tissues and the central nervous system. Much of our understanding of CPE function comes from two mouse models: *fat/fat* and *Cpe* knockout mice. *fat/fat* mice, which have a naturally-occurring point mutation (Ser202Pro) inactivating *Cpe*, have slowly developing, adult-onset obesity with hyperproinsulinaemia and infertility [4]. Complete knockout of *Cpe* causes a similar phenotype, although subtle differences have been reported in weight that may be due to strain and/or housing differences [11].

The obesity seen in these mouse models is due to an increased consumption of food, reduced basal metabolic rate, reduced utilisation of lipids for energy and reduced spontaneous activity [11, 30]. CPE is involved in energy homeostasis through processing of a number of peptides known to have an anorexogenic effect, including α-melanocyte-stimulating hormone (α-MSH). CPE removes C-terminal residues from processing intermediates formed by the cleavage of pro-opiomelanocortin (POMC) by prohormone convertases 1 (PC1/3) and 2, to generate α-MSH. α-MSH activates the melanocortin-4 receptor (MC4R), exerting an anorexigenic and thermogenic effect [31]. Defects in endoproteolytic processing of POMC due to deficiency in PC1/3 or mutations in the *POMC* gene also cause monogenic severe obesity [32, 33]. Interestingly, thus far, more putative obesity-causing mutations have been found in the POMC region downstream of the α-MSH coding region, affecting only the β variant of MSH. This might indicate that β-MSH also plays an important role in the appetite pathway in humans [34, 35]. β-MSH is not directly processed by CPE, although detailed investigation of indirect pathways affecting its functioning levels in CPE deficiency has not been possible so far since mice do not express β-MSH [36, 37].

The absence of functional CPE in mice also directly impairs the processing of many other anorexigenic hormones and neuropeptides including cholecystokinin, proinsulin, and proglucagon [4, 7, 11, 38, 39]. Other anorexigenic neuropeptides at reduced levels in CPE deficiency include Cocaine- and Amphetamine-Regulated Transcript, prothyrotropin releasing hormone, oxytocin and neurotensin [7, 11, 40]. While some neuropeptides, such as neuropeptide Y, with an orexigenic function are also decreased, other orexigenic peptides maintain normal levels or are increased in *Cpe* deficient mice [7]. So even though both body weight-increasing as well as body weight-lowering peptides are affected in *Cpe* deficiency, the mouse models become obese. This is thought to be due to the severe disruption of the body weight-lowering peptides [7, 30, 41] Therefore, the homozygous null mutation in the *CPE* gene is a plausible explanation for the proband’s hyperphagia and obesity, with multiple hormones/neuropeptides likely to be involved in the pathogenesis.

Besides being obese, both mouse models also show slowly increasing glucose concentrations leading to hyperglycaemia, caused by obesity-associated insulin resistance and a lack of fully processed insulin and insulinotropic GLP-1. The diabetes is, however, age-dependent, and not seen in young or very old *fat/fat* mouse models, and is also gender and strain dependent [4, 11]. Thus, the proband’s T2DM is also potentially explained by the homozygous null *CPE* mutation.

Mouse models indicate that lack of CPE also has effects on bone remodelling, reproduction, neuroprotection, and behavioural anomalies including memory deficits, depression and anxiety like phenotypes [8–11, 40]. Although, no signs of depression or anxiety were present in the proband, her intellectual disability and hypogonadotrophic hypogonadism could be explained by the CPE deficiency, though other causes of these specific phenotypes cannot be definitively excluded. However, the similarity to the early onset-obesity and hypogonadotrophic hypogonadism seen in a woman with reduced PC1/3 activity, and the early-onset obesity, intellectual disability and hypogenitalism in the deceased brother of the proband investigated here does point to a likely homozygous genetic cause of these features [32]. Although gastrointestinal problems have been repeatedly described in PC1/3 deficient patients, such symptoms were not present in the proband [42, 43].

*Cpe* was identified as the causative gene in the *fat/fat* mouse two decades ago, around the same time leptin was identified as the missing hormone in the *ob/ob* mouse, and a mutation in its receptor identified in the *db/db* mouse [4]. However, unlike the subsequent identification of human mutations in *LEP* and *LEPR*, a causative null mutation in *CPE* has to our knowledge not been described before in humans. This reported case of a *CPE* knockout in humans demonstrates clear similarities in the observed phenotypes between humans and the *fat/fat* and *Cpe* knockout mice. This is only the third example in which congenital deficiency of a pro-hormone/peptide processing enzyme has been associated with human disease, the others being PC1/3 in human obesity (which is involved in overlapping endocrine/metabolic pathways) [32], and *PCSK9* in autosomal dominant hypercholesterolaemia [44].

The proband presented here exhibits hypogonadotrophic hypogonadism and intellectual disability, which may be diagnostic features of CPE deficiency and so genetic investigation of *CPE* is warranted in similar cases where other known genetic causes have been excluded, especially with co-existent obesity. Ongoing detailed phenotyping of the homozygote proband and heterozygote family members, including assessment of circulating levels of hormones regulating glycaemia and appetite regulation, will further clarify the role of the CPE pro-hormone/peptide processing enzyme in human physiology. Our data add to the growing list of monogenic obesity genes in humans, which will help provide diagnostic and therapeutic opportunities for this challenging and often clinically neglected group of patients.

## Web Resources

FastQC version 0.10.0: http://www.bioinformatics.babraham.ac.uk/projects/fastqc

BWA mem version 0.7.2: http://arxiv.org/abs/1303.3997?context=q-bio

hs37d5ss decoy sequences from 1000 genomes project: ftp://ftp.1000genomes.ebi.ac.uk/vol1/ftp/technical/reference/phase2_reference_assembly_sequence/README_human_reference_20110707 and

ftp://ftp.1000genomes.ebi.ac.uk/vol1/ftp/technical/reference/phase2_reference_assembly_sequence/hs37d5.fa.gz

Picard tools version 1.85: http://picard.sourceforge.net/

## Supporting Information

S1 Table Overview of obesity genes. (XLSX)

S2 Table Overview of intellectual disability genes. (XLSX)

S3 Table Threshold cycle and ΔΔCt values for the *CPE* and *HPRT* assays. (DOCX)
