# Supplementary material for: Truncating Homozygous Mutation of Carboxypeptidase E (CPE) in a Morbidly Obese Female with Type 2 Diabetes Mellitus, Intellectual Disability and Hypogonadotrophic Hypogonadism
Source: PLoS One. 2015 Jun 29;10(6):e0131417. doi: 10.1371/journal.pone.0131417 (PMC4485893; doi:10.1371/journal.pone.0131417)
Supplement: S3 Table — (DOCX) [file pone.0131417.s003.docx]

| Sample | Ct for *HPRT* product | Ct for *CPE* product | ΔΔCt |
| --- | --- | --- | --- |
| Ctrl 1 | 27.52 | 35.28 | 2.12 |
| Ctrl 2 | 28.98 | 34.48 | -0.14 |
| Ctrl 3 | 29.80 | 33.61 | -1.83 |
| Ctrl 4 | 30.83 | 36.31 | -0.16 |
| Ctrl 5 | 28.67 | 34.02 | -0.29 |
| Ctrl 6 | 29.02 | 35.54 | 0.88 |
| Patient | 30.17 | No amplification | N/A |
| Sibling | 28.66 | 34.81 | 0.51 |
| Reference | 29.00 | 34.64 | N/A |

**S3 Table: Threshold cycle and ΔΔCt values for the *CPE* and *HPRT* assays**

Ct, threshold cycle; higher values indicate lower transcript levels. Mean Ct values of triplicates are given, except for Ctrl 1, in which the Ct of duplicates is given (one failed to amplify).
